# Centrioles initiate cilia assembly but are dispensable for maturation and maintenance in *C. elegans*

**DOI:** 10.1083/jcb.201610070

**Published:** 2017-06-05

**Authors:** Daniel Serwas, Tiffany Y. Su, Max Roessler, Shaohe Wang, Alexander Dammermann

**Affiliations:** 1 Max F. Perutz Laboratories, University of Vienna, Vienna Biocenter (VBC), A-1030 Vienna, Austria; 2 Ludwig Institute for Cancer Research, Department of Cellular and Molecular Medicine, University of California, San Diego, La Jolla, CA 92093

## Abstract

Cilia are cellular projections that assemble on centriole-derived basal bodies. While cilia assembly is absolutely dependent on centrioles, it is not known to what extent they contribute to downstream events. The nematode *C. elegans* provides a unique opportunity to address this question, as centrioles do not persist at the base of mature cilia. Using fluorescence microscopy and electron tomography, we find that centrioles degenerate early during ciliogenesis. The transition zone and axoneme are not completely formed at this time, indicating that cilia maturation does not depend on intact centrioles. The hydrolethalus syndrome protein HYLS-1 is the only centriolar protein known to remain at the base of mature cilia and is required for intraflagellar transport trafficking. Surprisingly, targeted degradation of HYLS-1 after initiation of ciliogenesis does not affect ciliary structures. Taken together, our results indicate that while centrioles are essential to initiate cilia formation, they are dispensable for cilia maturation and maintenance.

## Abstract (precis)

Centrioles are known to be essential for cilia assembly. However, their contribution has not been clearly defined. Serwas et al. show that centrioles degenerate early in *C. elegans* ciliogenesis. Ciliary structures are not completely formed at this time, indicating that cilia maturation does not depend on intact centrioles.

## Introduction

Cilia are microtubule-based projections found on the surface of many eukaryotic cells. Present in numbers ranging from one or two to several hundred, motile cilia enable the movement of cells or fluid over the surface of cells. While motile cilia can also be sensory, nonmotile primary cilia, a metazoan innovation, are specialized for signal transduction and function in the perception of environmental stimuli and cell signaling (Satir and Christensen, 2007). The widespread importance of cilia in vertebrates is underlined by the pleiotropic nature of human cilia-related disorders or ciliopathies, with clinical manifestations including situs inversus, respiratory dysfunction, infertility, and hydrocephalus for disorders affecting motile cilia. Disorders affecting nonmotile cilia are characterized by an even wider array of phenotypes, including defects in neural tube development and limb patterning, cystic kidney, liver and pancreatic diseases, retinal degeneration, anosmia, cognitive defects, and obesity (Badano et al., 2006). Ciliopathies nevertheless represent only the weak end of the phenotypic spectrum, with complete loss of cilia resulting in embryonic lethality in mice (Murcia et al., 2000).

Except in rare and unusual circumstances (e.g., Dallai et al., 2016), cilia arise from centriole-derived basal bodies, which impart on them their characteristic ninefold symmetry. Ciliogenesis initiates with the translocation of centrioles to the cell surface where they are anchored via appendages (the so-called transition fibers) present at their distal ends (Schmidt et al., 2012). Formation of the outer microtubule doublets of the axoneme that constitute the core of the cilium occurs by direct extension of centriolar microtubules, whereas the central pair of microtubules present in motile cilia forms independently distal to the basal body (O’Toole et al., 2003). Separating the basal body from the cilium proper is the transition zone, an elaborate structure characterized by Y-links connecting axonemal microtubules to the ciliary membrane, which serves to restrict protein access, establishing a distinct cellular compartment (Reiter et al., 2012). Extension of the axoneme within this compartment requires bidirectional microtubule motor-driven intraflagellar transport (IFT) which delivers cargo to the growing tip (Kozminski et al., 1993). Essential for their initial assembly, IFT continues to occur in most mature cilia and is required for their maintenance and function, though the extent to which the axoneme itself is sensitive to IFT perturbation depends on the rate of tubulin turnover at the ciliary tip (Marshall and Rosenbaum, 2001; Hao et al., 2011; San Agustin et al., 2015; Fort et al., 2016).

As the platform for assembly of key ciliary structures including the transition fibers and axoneme, centrioles are clearly essential for ciliogenesis. Failure to form centrioles invariably results in failure to form cilia (Basto et al., 2006; Azimzadeh et al., 2012). However, to what extent centrioles contribute to subsequent events in cilia assembly and maintenance remains unclear. This is a difficult question to address given the exceptional stability of centrioles; once formed, centrioles exhibit no appreciable cytoplasmic exchange of tubulin or other structural components (Kochanski and Borisy, 1990; Kirkham et al., 2003; Leidel and Gönczy, 2003; Leidel et al., 2005) and remain unchanged in their molecular composition over many cell divisions (Balestra et al., 2015). In the absence of any known factors specifically required for their maintenance, centrioles can therefore currently not be eliminated subsequent to their formation by molecular means such as RNAi-mediated depletion of key proteins.

Here, we take advantage of the natural degeneration of basal bodies in *Caenorhabditis elegans* sensory neurons to define the role of centrioles in ciliogenesis. By examining animals at different stages of neuronal differentiation by electron tomography and fluorescence microscopy using a range of centriolar and ciliary markers, we find that loss of centriolar structures and structural components precedes transition zone expansion and axoneme extension. Centrioles are therefore dispensable for cilia maturation and maintenance.

## Results

### Centrioles do not persist at the base of mature *C. elegans* cilia

**Figure 1.**
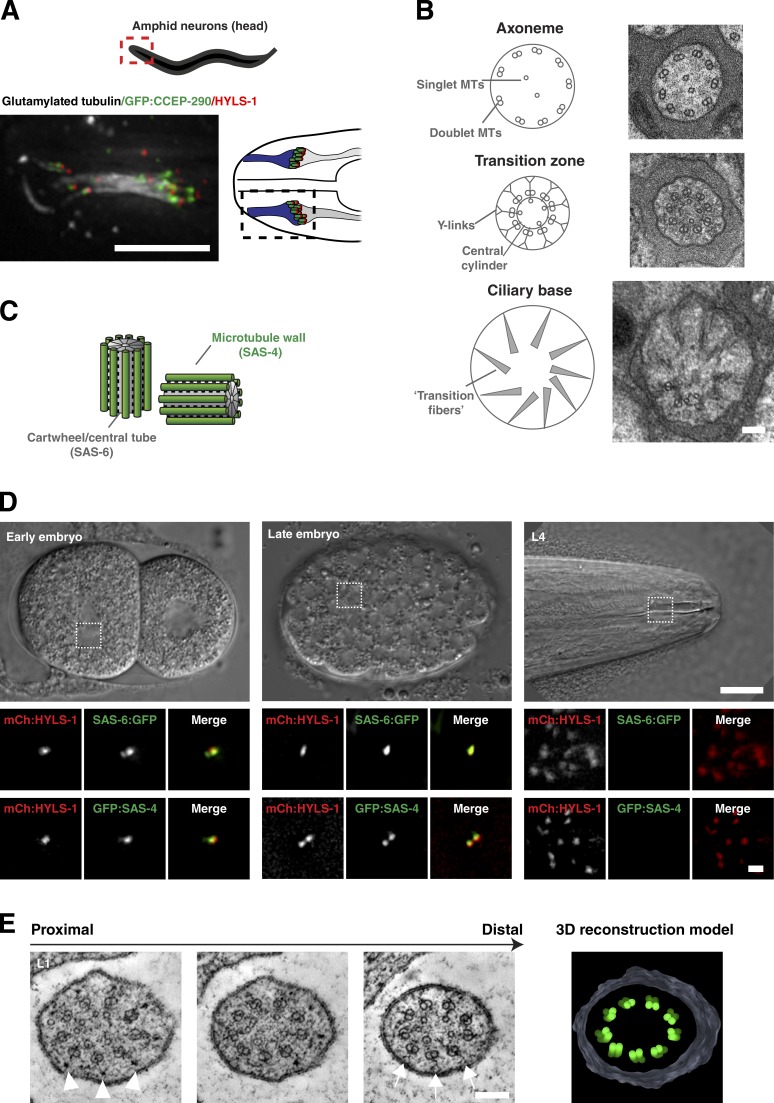
**Centrioles are not present at the base of mature *C. elegans* cilia.** (A) Immunofluorescence micrograph of amphid cilia in L4-stage larva expressing GFP:CCEP-290 (transition zone) and stained for HYLS-1 (ciliary base) and glutamylated tubulin (axoneme). 10 cilia form a bundle that is in contact with the external environment via a channel formed by the sheath and socket glia cells. Note that cell bodies are located ∼100 µm from the ciliary base and, therefore, outside the field of view. (B) Transmission electron micrographs of L4-stage amphid cilium at the level of the proximal segment of the axoneme, transition zone, and ciliary base with explanatory schematics. Morphologically recognizable centriole/basal body structures are missing from the ciliary base. MTs, microtubules. (C) Schematic of centriole pair in *C. elegans* early embryo. SAS-6 is a component of the cartwheel/central tube, whereas SAS-4 is associated with the outer microtubule wall. (D) SAS-6 and SAS-4 are not present at the base of mature cilia. Panels show centrioles in two-cell and gastrula-stage embryos and ciliary base in L4-stage larvae of a strain coexpressing GFP:SAS-4/SAS-6:GFP and mCherry:HYLS-1 (mCh:HYLS-1). (E) Tomographic slices and 3D reconstruction model of the ciliary base of L1-stage amphid cilium. Axonemal microtubules continue proximally as cilium widens at the base. Note additional electron densities between doublet microtubules and cell membrane (arrowheads). Transition zone elements including Y-links (arrows) can be seen in more distal planes. See also Videos 1 and 2. Bars: (A) 5 µm; (B) 200 nm; (D, insets) 1 µm; (D) 10 µm; (E) 100 nm.

The centriole-derived basal body is a near-invariant feature at the base of mature cilia. Not so in *C. elegans*. Cilia in the worm are exclusively found in postmitotic sensory neurons located in the head and tail of the animal (Fig. 1 A; Inglis et al., 2007). Examination of these cilia by transmission electron microscopy reveals a canonical axoneme, composed of nine outer doublet microtubules, as well as a variable number of inner singlet microtubules and transition zone, with characteristic Y-links connecting doublet microtubules to the ciliary membrane, as well as a central cylinder also found in other organisms (Fig. 1 B; Perkins et al., 1986; Schouteden et al., 2015). However, proximal to the transition zone, *C. elegans* cilia lack morphologically recognizable basal bodies. Instead, cilia terminate in apparent connections from the peripheral doublets to the cell membrane reminiscent of the transition fibers in other organisms (Fig. 1 B; Perkins et al., 1986).

Centriolar proteins likewise fail to localize to the base of mature cilia. Of the conserved machinery underlying centriole assembly, two proteins, SAS-6 and SAS-4, stably incorporate into centrioles as they form (Kirkham et al., 2003; Leidel and Gönczy, 2003; Leidel et al., 2005) as structural components of the cartwheel/central tube and centriolar microtubule wall, respectively (Fig. 1 C; Dammermann et al., 2008; Kitagawa et al., 2011; van Breugel et al., 2011). Neither protein is detectable by immunofluorescence in late-stage embryos or the adult worm (Dammermann et al., 2009 and not depicted; see also below). To exclude potential artifacts caused by antigen inaccessibility, we confirmed protein localization using GFP fusions expressed under endogenous regulatory sequences. Importantly, these fusions not only fully restored viability of *sas-4*/*sas-6*–null mutants, but also sustained proper cilia assembly as determined using the dye-fill assay for cilia structural integrity (Fig. S1, A and B; Hedgecock et al., 1985). In contrast to centrosomes in the mitotically dividing embryo, no GFP signal could be observed at the ciliary base in larvae or the adult worm (Fig. 1 D). However, as previously noted, HYLS-1, a protein recruited by SAS-4 and stably incorporated during centriole assembly, remains at the ciliary base (Fig. 1 D; Dammermann et al., 2009).

A recent reexamination of *C. elegans* sensory architecture by serial section electron tomography provided evidence that what had originally been described as transition fibers (Fig. 1 B; Perkins et al., 1986) are largely an artifact of projecting splayed microtubules at the ciliary base in transmission electron micrographs (Doroquez et al., 2014). Our own electron tomograms of the base of mature cilia using both high-pressure freezing/freeze substitution and chemical fixation support this (Fig. 1 E and Videos 1 and 2). The outer doublet microtubules of the axoneme continue proximally and splay apart as the cilium widens below the transition zone. The inner singlet microtubules also extend proximally. Transition zone elements including the central cylinder and Y-links are not observed in proximal planes. However, there are also no clear indications of centriolar/basal body structures (cartwheel/central tube, appendages), although accumulations of electron-dense material can be observed between doublet microtubules and the plasma membrane (Fig. 1 E, arrowheads). In summary, light and electron microscopy analysis of mature *C. elegans* cilia indicates that centrioles indeed degenerate, and core structural components are lost.

### Centrioles degenerate early in neuronal differentiation

Next, we sought to examine at what stage of neuronal differentiation centrioles are lost. Reconstructing the timeline of centriole degeneration and concomitant cilia assembly is aided by the highly stereotypical nature of *C. elegans* development: the timing and pattern of cell divisions and the relative position of cells is essentially unchanged from animal to animal (Sulston et al., 1983), as is the timing of individual cell-cycle events (Oegema and Hyman, 2006; Begasse et al., 2015). This invariance greatly facilitates examination of multiple markers alongside ultrastructural analysis at different stages of differentiation. For our analysis, we decided to focus on the amphids, the primary sensory organ in the head of the animal, easily identified and examined by both light and electron microscopy.

**Figure 2.**
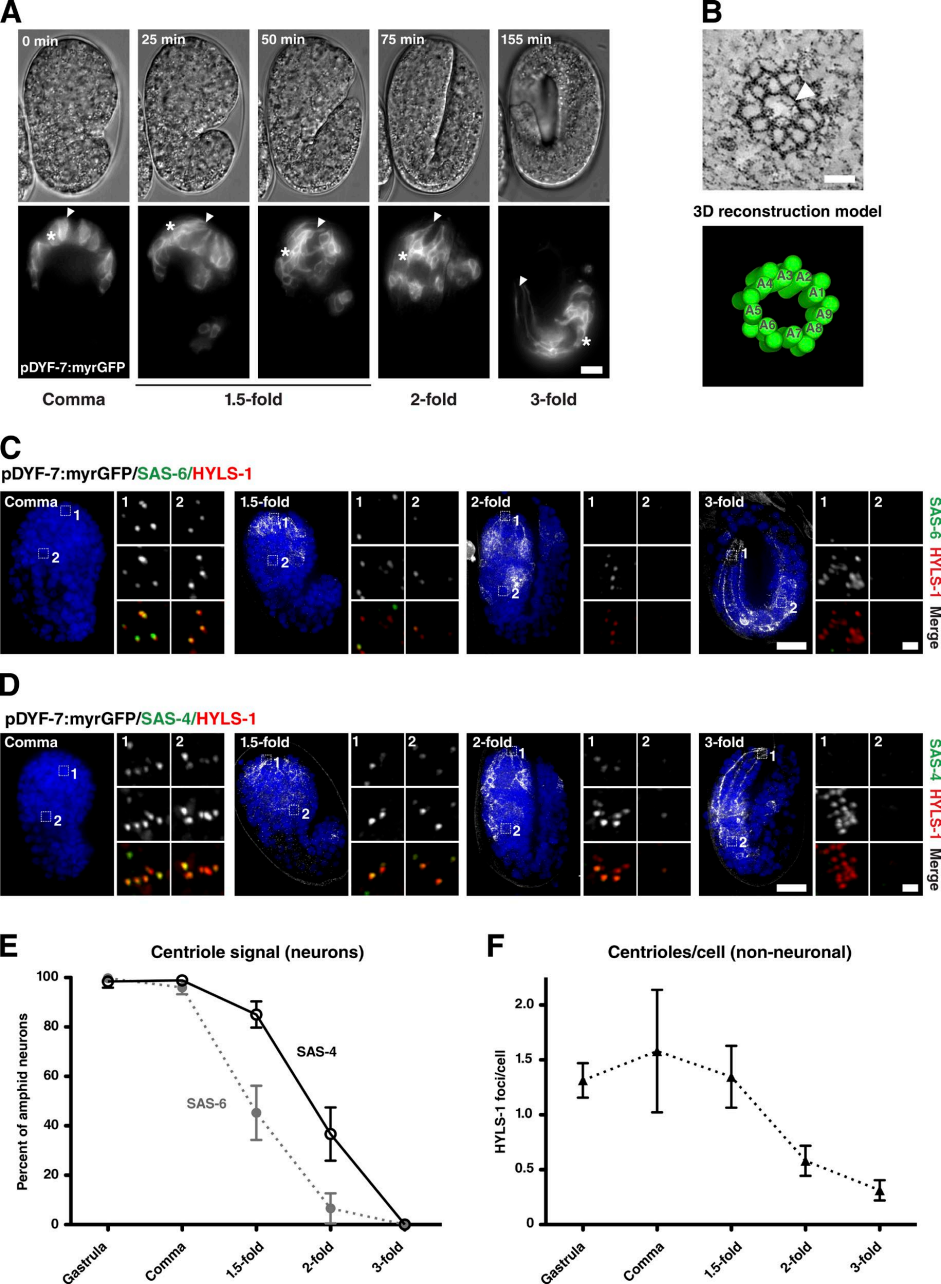
**Centrioles are lost early in neuronal differentiation.** (A) Stills from time-lapse video of embryo expressing myristoylated GFP (myrGFP) in amphid neurons undergoing retrograde migration. DIC and single-plane GFP images taken at the indicated time intervals corresponding to comma, 1.5-fold, twofold, and threefold stages. Note the position of dendritic tips (arrowheads) remains fixed as cell bodies (asterisks) move. At the threefold stage, the worm begins to move inside the egg because of muscle contraction. See also Video 3. (B) Tomographic slice and 3D reconstruction model of centriole in a myristoylated GFP–positive neuron of a comma-stage embryo. Note the centriole displays doublet microtubules. Electron densities at the center of the centriole may represent elements of the central tube/cartwheel (arrowhead). See also Video 4. (C and D) Immunofluorescence micrographs of comma-, 1.5-fold–, twofold-, and threefold-stage embryos expressing myristoylated GFP in amphid neurons and stained for HYLS-1 and SAS-6 (C) or SAS-4 (D). Insets show magnified view of ciliary base (1) and nonneuronal cells elsewhere in the head of the embryo (2). Centriolar signal is lost from the ciliary base, with loss of SAS-6 preceding that of SAS-4, while HYLS-1 remains. All three proteins are lost in nonneuronal cells coinciding with terminal differentiation. (E and F) Quantitation of centriolar signal in amphid neurons (percentage of HYLS-1 foci positive for SAS-6 or SAS-4; E) and nonneuronal cells (HYLS-1 foci per nucleus; F) from images as in C and D. Error bars are 95% confidence intervals. *n* = 5–9 animals per condition. Bars: (A, C, and D) 10 µm; (B) 50 nm; (C and D, insets)1 µm.

The neuroblasts that give rise to anterior neurons including the amphids complete their terminal cell divisions after the end of gastrulation, between 330 and 450 min after fertilization (times at 22°C). Anterior neurons move toward the tip of the head and then posterior again, laying down their dendrites as they go, a process termed retrograde extension (Fig. 2 A and Video 3; Heiman and Shaham, 2009). The embryo elongates and becomes thinner because of contraction of the hypodermis from the so-called comma stage (430 min after fertilization) through the 1.5-fold (460 min), twofold (490 min), and threefold (550 min) stages (Fig. 2 A). The worm eventually hatches at 840 min after fertilization (Sulston et al., 1983).

Shortly after terminal cell division at the comma stage, centrioles are still clearly present, as assessed by SAS-4 and SAS-6 signal coincident with HYLS-1 in neurons marked by Pdyf-7 neuronal promoter-driven GFP (Fig. 2, C and D). Interestingly, these centrioles appear to be composed of doublet microtubules (Fig. 2 B and Video 4), in contrast to the singlet microtubule centrioles found in sperm and the early embryo (Wolf et al., 1978; Pelletier et al., 2006). Over the course of the next hour from the comma to twofold stage, SAS-4 and SAS-6 signal declines dramatically, with loss of the cartwheel component SAS-6 preceding that of the centriolar microtubule wall component SAS-4 (Fig. 2, C–E). Dynamically localized centriolar components ZYG-1, SPD-2, and SAS-5 are likewise lost with a timing similar to or preceding SAS-6 (Fig. S2). In contrast, HYLS-1 signal remains largely unchanged and is used as a reference point for this analysis. Centriole degeneration is not unique to neuronal differentiation. Indeed, there is a marked loss of centriolar protein signal (in nonneuronal cells also including HYLS-1) across the entire embryo at the same time (Fig. 2, C–F), which coincides with the terminal divisions of most of the cells giving rise to the *C. elegans* soma (Sulston et al., 1983). Centriole degeneration then occurs relatively early during neuronal differentiation, raising the question how far cells have progressed in ciliogenesis at this stage.

### Formation of ciliary structures

#### Transition zone assembly

The transition zone is one of the most striking and characteristic features of the cilium. At 1 µm in length compared with ∼300 nm in vertebrates, the transition zone is rather well developed in *C. elegans* and appears to contain periodic elements which may correspond to stacked Y-links (Lambacher et al., 2016). While the molecular architecture of the transition zone remains to be fully elucidated, increasing evidence points to it being organized into multiple protein complexes or modules, recruited independently of each other and with distinct localization patterns and functions (Garcia-Gonzalo et al., 2011; Sang et al., 2011; Williams et al., 2011; Chih et al., 2012; Basiri et al., 2014; Schouteden et al., 2015; Pratt et al., 2016; Vieillard et al., 2016). Of these, the proteins of the MKS and NPHP modules localize to the transition zone periphery where they cooperate in the formation of Y-links (Williams et al., 2011; Yang et al., 2015; Lambacher et al., 2016). The contribution of CEP290 and RPGRIP1L is less clear, but their location at the proximal and distal core of the transition zone, respectively (Yang et al., 2015), supports a role in scaffolding and anchoring transition zone structures (Basiri et al., 2014; Schouteden et al., 2015; Li et al., 2016).

**Figure 3.**
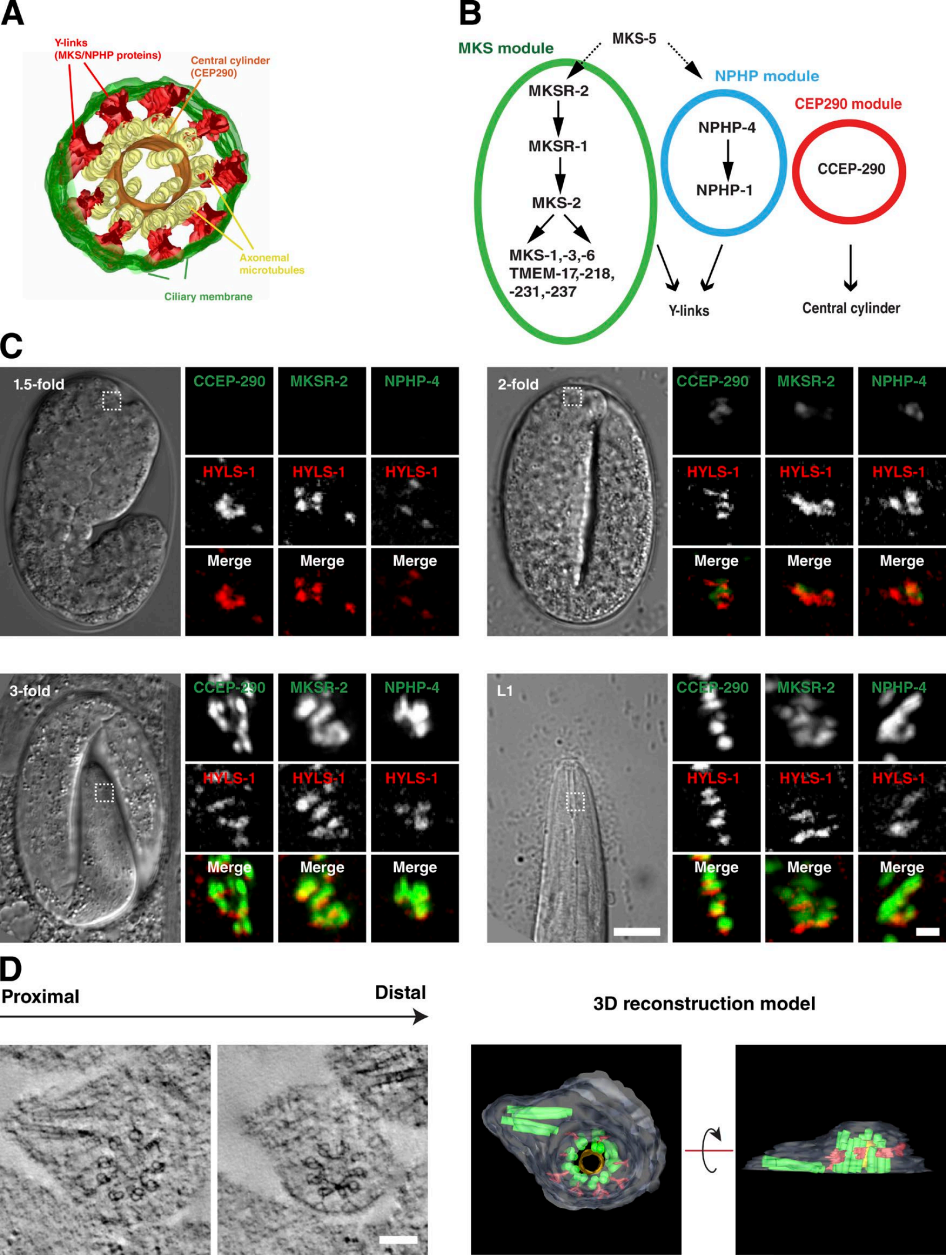
**Transition zone assembly.** (A and B) 3D reconstruction model (A) and molecular assembly hierarchies (B) for the *C. elegans* transition zone based on Schouteden et al. (2015) (A) and Huang et al. (2011), Williams et al. (2011), Jensen et al. (2015), Roberson et al. (2015), Schouteden et al. (2015), and Li et al. (2016) (B). CCEP-290 is an essential component of the central cylinder, whereas MKS and NPHP module components function in assembly of Y-links. MKSR-2 and NPHP-4 are upstream components in the MKS and NPHP assembly pathways, respectively. (C) Recruitment of transition zone components to the site of cilia assembly marked by HYLS-1. Panels show 1.5-fold–, twofold-, and threefold-stage embryos and L1-stage larvae coexpressing GFP:CCEP-290, MKSR-2, NPHP-4, and mCherry:HYLS-1. Initial recruitment of all three components occurs at the twofold stage, with signal reaching maximal levels at the threefold stage. (D) Tomographic slices and 3D reconstruction model of the ciliary base of twofold-stage amphid cilium. A pair of centrioles can be seen, one of which is decorated by a single array of Y-links and a discontinuous central cylinder and enveloped by the ciliary membrane. Note marked widening of the centriole toward the base. See also Video 5. Bars: (C) 10 µm; (C, insets) 1 µm; (D) 100 nm.

To examine the timing of transition zone assembly, we chose MKSR-2 and NPHP-4 as upstream components of the MKS and NPHP module assembly pathways involved in Y-link formation (Williams et al., 2011) and CCEP-290 as a putative central cylinder component (Fig. 3, A and B; Schouteden et al., 2015). Endogenous promoter GFP fusions were validated by their ability to rescue the respective null mutant in the dye-fill assay (Fig. S1 C). Recruitment of these proteins to the site of cilia assembly was then assessed using mCherry:HYLS-1 as a marker. For all three proteins, initial recruitment occurs at the twofold stage (Fig. 3 C), a stage at which SAS-4 but not SAS-6 is still present (compare Fig. 2, C–E). Levels subsequently increase, with the transition zone expanding to near-adult dimensions by the threefold stage (Fig. 3 C). Examination of twofold stage embryos by electron tomography revealed centrioles docked at the cell surface, decorated at their distal end with a single array of Y-links and an incomplete central cylinder (Fig. 3 D and Video 5). Consistent with the rather minimal signal for CCEP-290, MKSR-2, and NPHP-4 at this time, the transition zone does not yet extend significantly in length, with the incipient cilium capped within 100 nm of the centriole by the ciliary membrane. While the centriole itself still appears reasonably intact, there is already a significant widening at the proximal end, potentially reflecting the loss of SAS-6–containing inner centriolar structures. Also notable is an apparent daughter centriole, oriented orthogonally, not found in mature cilia.

In summary, transition zone proteins begin to be recruited and key elements (Y-links and central cylinder) start to assemble at a time when centrioles are still present. However, transition zone expansion and axoneme extension occur at later stages, after loss of centrioles and centriolar proteins.

#### IFT-dependent axoneme extension

Axoneme extension beyond the transition zone involves microtubule motor-driven transport of IFT trains assembled from IFT-A and IFT-B subcomplexes delivering subunits to the ciliary tip (Cole et al., 1998). In *C. elegans*, anterograde transport is mediated by two kinesin-2 motors, heterotrimeric kinesin-II and homodimeric OSM-3. Both motors cooperate to form the proximal segment of the axoneme, composed of nine doublet microtubules, whereas assembly of the distal segment, composed of nine singlets, depends on OSM-3 alone (Snow et al., 2004). IFT docking at the ciliary base has been shown to require DYF-19/Fbf1, a protein associated with transition fibers in vertebrates (Wei et al., 2013).

**Figure 4.**
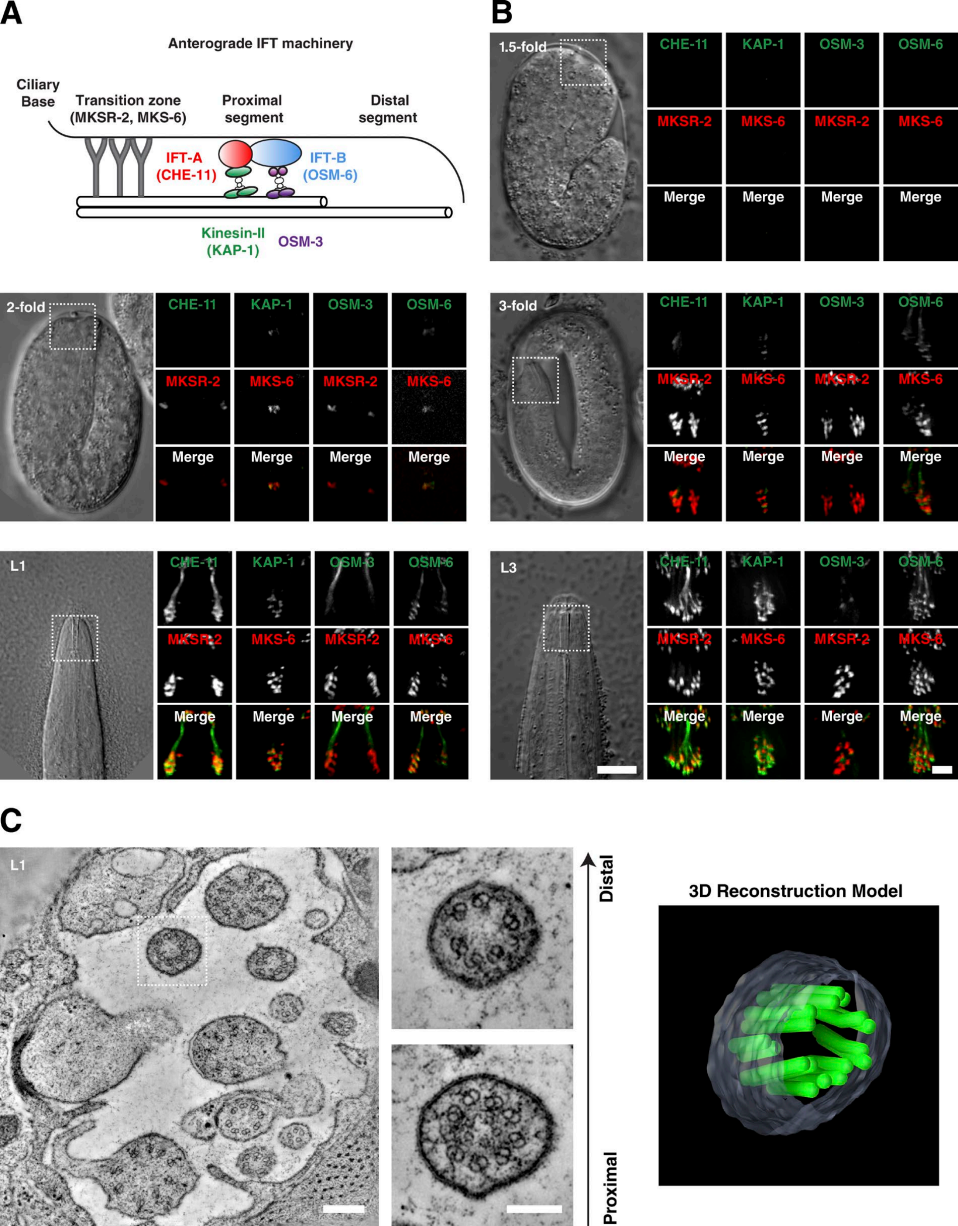
**IFT-dependent axoneme extension.** (A) Schematic of anterograde IFT machinery in *C. elegans*. Assembly of the proximal segment depends on the coordinated transport of IFT trains by kinesin-II and OSM-3 together, while assembly of the distal segment depends on OSM-3 alone. (B) Recruitment of fluorescently tagged IFT components (green in merge) to the site of cilia assembly marked by the transition zone components MKS-6 and MKSR-2 (red). Panels show 1.5-fold–, twofold-, and threefold-stage embryos and L1- and L3-stage larvae coexpressing CHE-11:mCherry, KAP-1:GFP, OSM-3:mCherry, or OSM-6:GFP and GFP:MKSR2 or MKS-6:mCherry as indicated. Initial recruitment of KAP-1 and OSM-6 occurs at the twofold stage of CHE-11 and OSM-3 at the threefold stage. IFT trafficking is only observed at L1. (C) Tomographic slice through base of the amphid channel in L1 larva, with magnified view and 3D reconstruction model of a single cilium at the level of the transition zone and proximal axoneme. Note cilium appears to terminate close to the transition zone. See also Video 6. Bars: (B) 10 µm; (B, insets) 1 µm; (C) 200 nm; (C, insets) 100 nm.

To examine IFT-dependent axoneme extension during *C. elegans* development, we chose CHE-11 and OSM-6 as representatives of the IFT subcomplexes A and B, respectively, and KAP-1 (kinesin-II) and OSM-3 for the two anterograde motors (Fig. 4 A). For each protein, endogenous promoter fluorescent fusions were validated by mutant rescue where possible (*kap-1* mutants do not present an easily detectable phenotype; Fig. S1 D). We then monitored recruitment using the transition zone marked by MKSR-2 and MKS-6 as a point of reference (Fig. 4 B). While some IFT proteins (KAP-1 and OSM-6) were observed as early as the twofold stage, significant accumulations of all four proteins were only observed after hatching at the first larval stage (L1). Coincident with initial IFT recruitment, DYF-19 was found to localize to the ciliary base from the twofold stage (Fig. S3 A). Consistent with the lack of axoneme extension beyond the transition zone observed by electron microscopy in twofold-stage embryos, any early IFT signal largely overlaps with the transition zone. Only from the L1 stage onwards are IFT proteins clearly seen distal to the transition zone.

In mature *C. elegans* amphids, 10 cilia extend into a channel formed by the sheath and socket glia cells through which they are in contact with the external medium. With their transition zones embedded in the matrix at the base of the sheath channel, the proximal segment of the axoneme extends 4 µm from the transition zone, whereas the singlet microtubules of the distal segment continue for another 2.5 µm (Fig. 4 A; Perkins et al., 1986; Doroquez et al., 2014). Electron tomography of the base of the amphid channel in L1 larvae show the expected number of cilia. However, while some cilia are already fully developed, others terminate close to the transition zone, with doublet microtubules extending to different lengths. These cilia further lack a clear distal segment of singlet microtubules (Fig. 4 C and Video 6). Axoneme extension then is a gradual process, largely occurring during the larval stages.

### The centriole remnant: HYLS-1 function reexamined

While centrioles largely degenerate and core centriolar structural components are lost, one centriolar protein, HYLS-1, remains at the ciliary base. Like SAS-4, HYLS-1 is stably incorporated into centrioles as they form, as a component associated with the centriolar microtubule wall. Unlike SAS-4, HYLS-1 is not required for centriole assembly or stability, or the ability of centrioles to form centrosomes. Instead, HYLS-1 is specifically required for centrioles to form cilia (Dammermann et al., 2009). Loss of HYLS-1 results in severe defects in axoneme assembly and transition zone organization, at least in part because of impaired entry of ciliary components including the IFT machinery via DYF-19, a protein it recruits to the ciliary base (Wei et al., 2016). The centriole remnant to which HYLS-1 localizes may therefore play an ongoing role in cilia trafficking.

**Figure 5.**
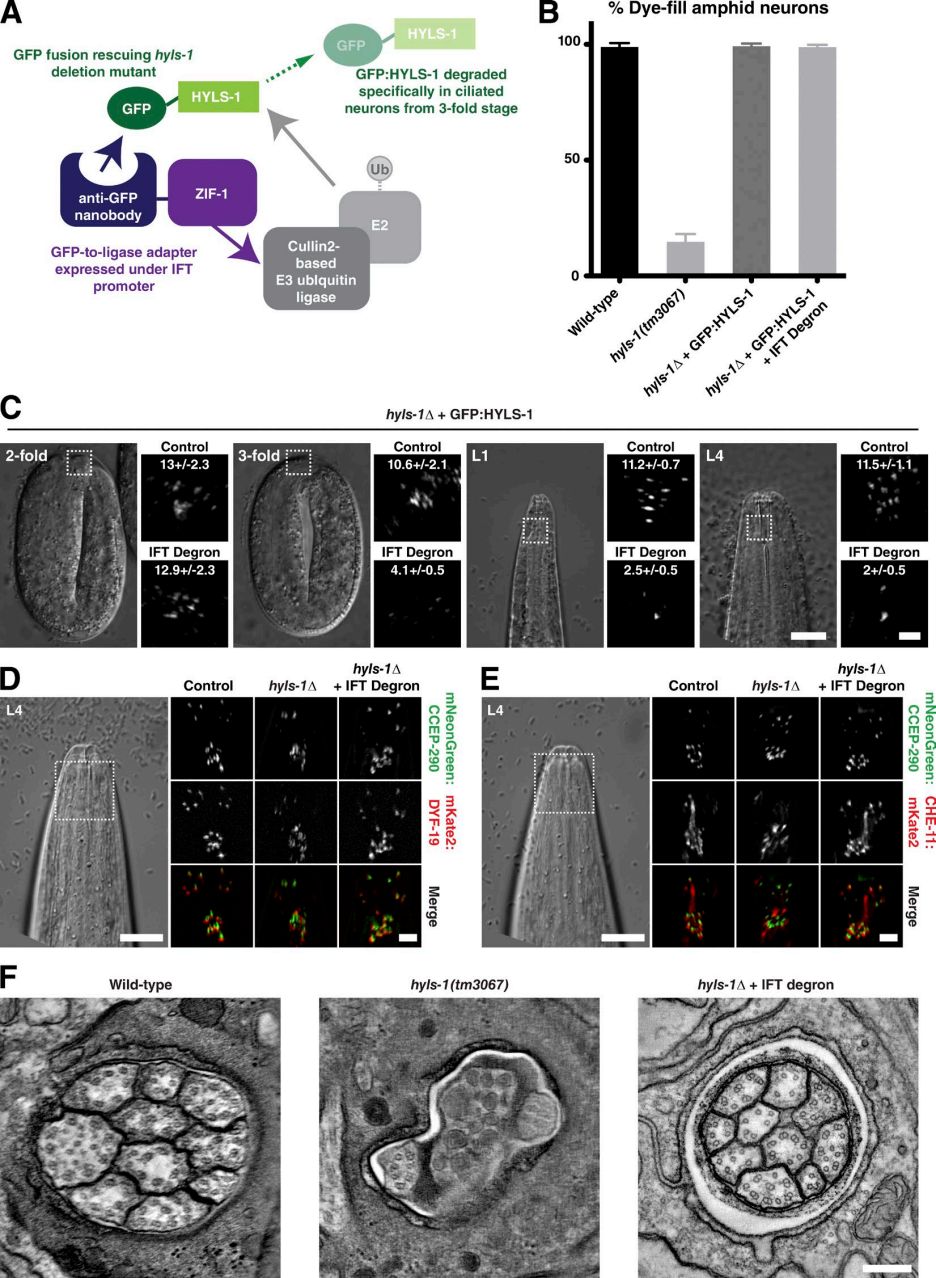
**HYLS-1 is dispensable for cilia maintenance.** (A) Schematic of the method used to degrade HYLS-1 specifically in ciliated neurons. (B) Amphid cilia structural integrity as assessed by dye filling. Dye-fill phenotype expressed as percentage of wild-type complement is shown. *hyls-1* mutants display strong defects because of compromised axoneme assembly. These are rescued by expression of GFP:HYLS-1 (Student’s *t* test; P < 0.0001). Degron-mediated degradation of GFP:HYLS-1 after onset of ciliogenesis does not impair dye filling (Student’s *t* test; P = 0.57). Error bars are 95% confidence interval. *n* > 50 animals per condition. (C) Loss of GFP:HYLS-1 because degron-mediated degradation occurs from the threefold stage. Control animals not expressing degron are shown for comparison. Some HYLS-1 foci remain in L4, presumably because of lack of expression of the degron in those neurons or inaccessibility of the GFP epitope. Number of foci/amphid bundle ± 95% confidence intervals (*n* = 5–15 amphids per condition) is shown. (D) *hyls-1* mutants display defects in targeting mKate2:DYF-19 to the ciliary base in amphid neurons. DYF-19 targeting is rescued in animals expressing GFP:HYLS-1 and the degron, despite late loss of HYLS-1. Transition zone marker mNeon:CCEP-290 is shown as a point of reference. (E) *hyls-1* mutants display defects in IFT trafficking, shown using CHE-11:mKate2. IFT trafficking is restored in animals expressing GFP:HYLS-1 and the degron. (F) Tomographic slice through the amphid channel in L4-stage larvae of wild type, *hyls-1* mutants, and *hyls-1* mutants expressing both GFP:HYLS-1 and the degron. The marked axoneme extension defects in *hyls-1* mutants are not observed in animals expressing both GFP:HYLS-1 and the degron. Bars: (C–E) 10 µm; (C, insets) 2 µm; (D and E, insets) 3 µm; (F) 100 nm.

To examine whether this is indeed the case, we sought to eliminate HYLS-1 after initiation of cilia assembly. To do this, we took advantage of the recently developed GFP nanobody::ZIF-1 degron system, which targets GFP-tagged proteins for ubiquitin-mediated degradation (Fig. 5 A; Wang et al., 2015). This degron was expressed under the Posm-6 IFT promoter, which is active in ciliated neurons from the threefold stage (Fig. 4 B). As continued neuronal gene expression could compromise efficacy of the degron, we used a Ppie-1 germline promoter-driven GFP:HYLS-1 transgene, which nevertheless fully rescues the *hyls-1*–mutant phenotype (Fig. 5 B; Dammermann et al., 2009). When combined with the IFT degron in an *hyls-1*–mutant background, GFP:HYLS-1 signal is observed at centrioles throughout the germline and in early embryos. HYLS-1 signal begins to decline at the threefold stage and is undetectable in most neurons by L1 (Fig. 5 C). Surprisingly, despite the loss of HYLS-1, cilia structural integrity remains intact as determined by the dye-fill assay (Fig. 5 B). In striking contrast, degradation of its target DYF-19 resulted in a *dyf-19*–null phenotype, despite a transient accumulation of the protein at the ciliary base (Fig. S3, B and C). To examine cilia morphology in more detail, we introduced degron-compatible mNeonGreen/mKate2 fusions to CCEP-290, DYF-19, and CHE-11 into the degron background. While DYF-19 fails to target to the ciliary base in *hyls-1* mutants (Wei et al., 2016), localization is unaffected by late loss of HYLS-1 (Fig. 5 D). Similarly, IFT entry is strongly perturbed in *hyls-1* mutants but apparently normal in degron worms (Fig. 5 E). Consistent with this, ultrastructural analysis reveals severe axonemal defects in *hyls-1* mutants but wild-type amphid morphology in degron worms (Fig. 5 F).

To conclude, HYLS-1 is required for the initial recruitment of DYF-19 and, thus, indirectly for cilia trafficking. However, while HYLS-1 continues to localize to the base of mature cilia, loss of HYLS-1 after onset of ciliogenesis does not perturb the ongoing protein trafficking which maintains the cilium.

## Discussion

**Figure 6.**
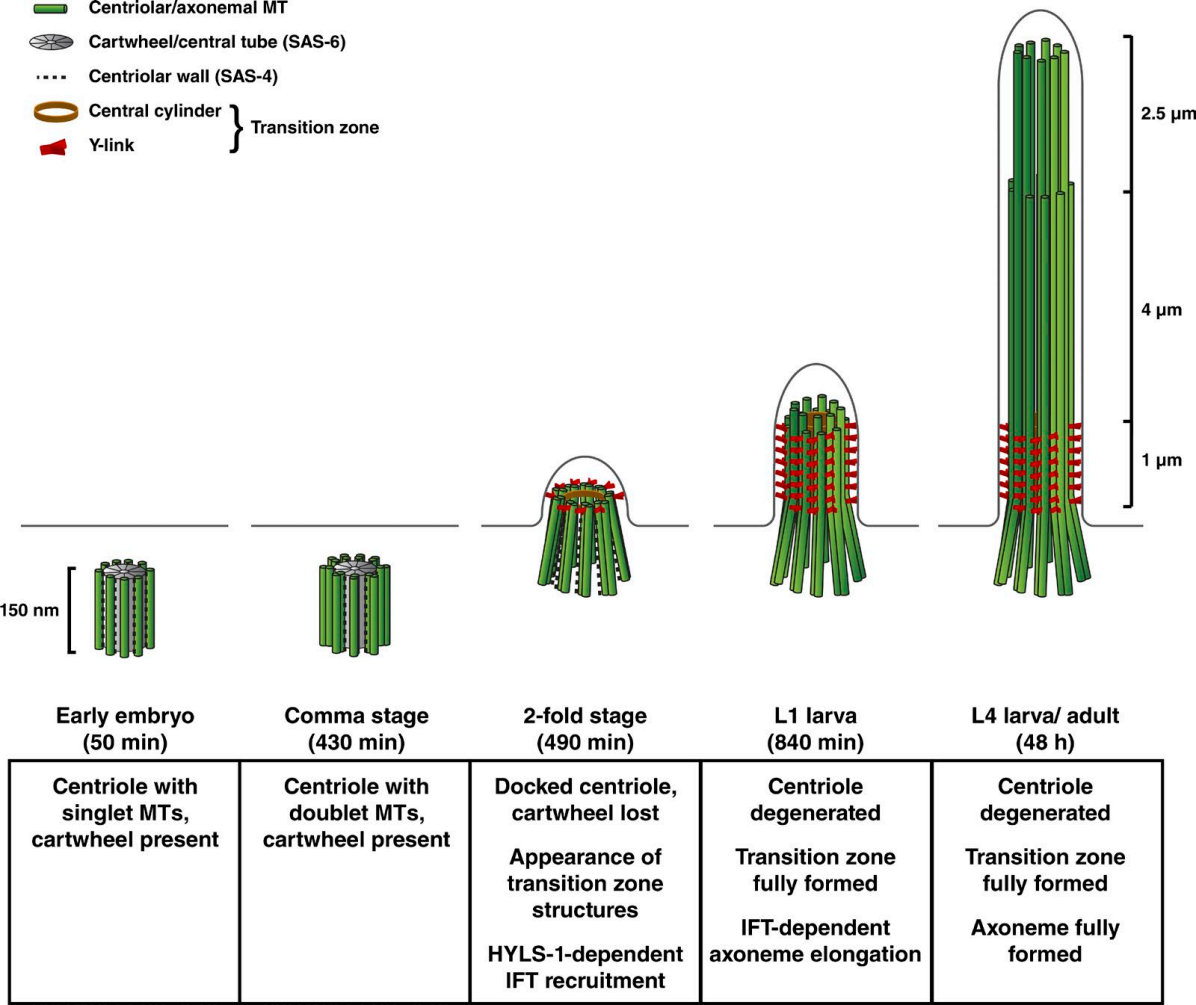
**A timeline for *C. elegans* ciliogenesis.** Centriole and ciliary architecture at key stages in the *C. elegans* life cycle. In contrast to the singlet centrioles in the early embryo (Wolf et al., 1978; Pelletier et al., 2006), centrioles in sensory neurons possess doublet microtubules (MTs). Within 1 h after terminal cell division (twofold stage; 490 min after fertilization), centrioles have docked to the plasma membrane and have begun assembling transition zone structures (Y-links and central cylinder). Expansion of transition zone structures requires another hour (threefold stage; 550 min; not depicted). IFT-dependent axoneme extension continues through the larval stages. Centriole degeneration begins with loss of the cartwheel/central tube (by twofold stage), followed by loss of the centriolar microtubule wall component SAS-4 (threefold stage). Centrioles are therefore not directly involved in later events including transition zone expansion and axoneme elongation. HYLS-1 remains on the centriolar remnant and is required for the initial DYF-19/Fbf1-dependent recruitment of IFT components. However, HYLS-1 is dispensable for continued IFT trafficking.

It has long been known that the *C. elegans* ciliary base lacks morphologically recognizable basal bodies (Perkins et al., 1986). Core centriolar structural components are likewise lost. Using a combination of fluorescence microscopy and electron tomography, we show that centriole degeneration occurs remarkably early during neuronal differentiation (Fig. 6).

Shortly after their final division, neurons still display canonical centrioles. However, as in *Drosophila* ciliated neurons (Gottardo et al., 2015), their wall is now composed of nine doublet microtubules rather than singlets, explaining the origin of the B-tubule of the ciliary axoneme. Within 1 h, this centriole docks at the cell membrane and is decorated by elements of the transition zone, including a set of Y-links and central cylinder. At this stage, inner centriolar structures marked by SAS-6 have already been lost, followed 1 h later by loss of the centriolar wall component SAS-4. Centrioles then are hollowed out from the inside, resulting in splaying apart of the microtubule wall, forming a remnant that was originally described as transition fibers (Perkins et al., 1986). Expansion of the transition zone and subsequent extension of ciliary axoneme occur after loss of centriolar structures and core centriolar structural components. While HYLS-1 continues to be associated with the centriole remnant, its role appears to be fulfilled early in ciliogenesis, in part by recruitment of DYF-19, a transition fiber component in vertebrates involved in IFT trafficking (Wei et al., 2013, 2016).

Our work therefore supports the notion that core centriolar structures merely initiate ciliogenesis, by providing a template for assembly of the axoneme and transition fibers, but play no direct part in downstream events. This indirect role could explain the paucity of interactions found between the core centriolar components SAS-4/CPAP and SAS-6 and ciliary proteins, compared with accessory structures, in particular the transition fibers (Gupta et al., 2015).

Loss of centrioles is not unique to *C. elegans* cilia. The basal body has been found to detach while the cilium persists for some time in certain green algae undergoing mitosis (Gaffal, 1977; Hoops and Witman, 1985). The basal body is therefore not required for structural maintenance and motility, at least in the short term. Partial or complete degeneration of the basal body has also been reported during spermatogenesis in both vertebrates and invertebrates (Manandhar et al., 2005). The lack of a structurally rigid centriole at the base of the axoneme may allow basal sliding (Vernon and Woolley, 2004), which has been proposed to be important for flagellar motility (Riedel-Kruse et al., 2007). In contrast, in *Tetrahymena*, the basal body needs to withstand mechanical stress generated by cilia motility and is required for cilia stability (Bayless et al., 2012). In the case of nonmotile *C. elegans* cilia, protected within the cuticle, stability may not be a factor. It is notable that centriole degeneration occurs not only in *C. elegans* ciliated neurons, but also throughout the animal as cells undergo terminal differentiation (Lu and Roy, 2014). In contrast to *C. elegans*, many terminally differentiated cells in vertebrates nevertheless retain the capacity to dedifferentiate and reenter the cell cycle, with disassembly of the primary cilium resulting in release of the basal body (Pugacheva et al., 2007). Just as the mitotic spindle acts as a basal body distributor (Friedländer and Wahrman, 1970), the primary cilium acts as a source of centrioles for cell division, a role not needed in developmentally fixed *C. elegans*.

In summary, our work indicates that while centrioles play an important role in initiating ciliogenesis, later events in cilia maturation (transition zone expansion and axoneme extension) and maintenance are independent of centrioles. The continued presence of basal bodies in other species may in part be a reflection of the continued reproductive capacity of the cells in which they are found.

## Materials and methods

### *C. elegans* strains and culture conditions

Strains used are listed in Table S1. Detailed information for mutants is available from WormBase (http://www.wormbase.org). Mutants were outcrossed six times to N2 wild type before phenotypic analysis and introduction of fluorescent markers. Transgenic strains expressing endogenous promoter fluorescent fusions for SAS-4, SAS-6 (Cabral et al., 2013), CHE-11, KAP-1, MKS-6, OSM-3, OSM-6 (Prevo et al., 2015), CCEP-290, MKSR-2, NPHP-4, and mCherry:HYLS-1 (Schouteden et al., 2015) and Ppie-1 germline promoter-driven GFP:HYLS-1 (Dammermann et al., 2009) were described previously. The strain expressing myristoylated GFP under the Pdyf-7 neuronal promoter at high transmission rates was derived from an existing extrachromosomal array strain (Heiman and Shaham, 2009) by UV irradiation, followed by outcrossing to N2 wild type. GFP:DYF-19 and GFP degron-compatible CHE-11:mKate2, mKate2:DYF-19, and mNeonGreen:CCEP-290 transgenes were expressed under endogenous regulatory sequences by cloning the corresponding genomic loci including N-/C-terminal fluorescent tag into the appropriate targeting vector and Mos1-mediated integration at a defined chromosomal locus (Frøkjær-Jensen et al., 2008, 2014). For targeted degradation of GFP:HYLS-1 and GFP:DYF-19 specifically in ciliated neurons, a GFP nanobody:ZIF-1 fusion (Wang et al., 2015), coexpressed with mCherry:histone H2B as a visible marker via an operon linker under an Posm-6 IFT promoter, was generated by Mos1 transposon insertion as above. All strains were maintained at 23°C.

### Immunofluorescence and fixed imaging

Immunofluorescence experiments were performed as previously described (Oegema et al., 2001) using Cy3/Cy5 directly labeled affinity-purified rabbit polyclonal antibodies against HYLS-1 (Dammermann et al., 2009), SAS-4, SAS-5, SAS-6, SPD-2, and ZYG-1 (Dammermann et al., 2004), as well as unlabeled mouse monoclonal antibodies against polyglutamylated tubulin (GT335; provided by C. Janke, Institut Curie, Paris, France). Residual fluorescence signal was used to detect myristoylated GFP protein in fixed embryos. In brief, embryos were permeabilized by freeze crack, fixed in −20°C methanol for 20 min, rehydrated in PBS, blocked for 20 min in AbDil (PBS, 2% BSA, 0.1% Triton X-100), incubated with directly labeled antibodies at 1 µg/ml (4 µg/ml GT335) in AbDil for 1.5 h, washed with PBST (PBS, 0.1% Triton X-100), incubated with secondary antibody (if applicable, 15 µg/ml Cy5-labeled donkey anti–mouse antibody; Jackson ImmunoResearch Laboratories, Inc.) in AbDil for 1 h, incubated with 1 µg/ml Hoechst 33342 in PBS for 5 min, washed again in PBST, and mounted in 0.5% p-phenylenediamine, 20 mM Tris, pH 8.8, and 90% glycerol. 0.2-µm 3D wide-field datasets were acquired using an Olympus 100× 1.4 NA Super Plan Apochromat lens on a DeltaVision microscope (Applied Precision) equipped with a 7-Color SSI module and CoolSNAP-HQ2 cooled charged-coupled device (CCD) camera (Photometrics), computationally deconvolved using the enhanced ratio constrained iterative deconvolution algorithm, and maximum intensity projected in SoftWorx (Applied Precision), before being imported into Adobe Photoshop for panel preparation. Exposure conditions and scaling were kept constant when comparing signal across different developmental stages. No nonlinear gamma corrections were applied during image processing.

### Live imaging

For live imaging, worms were anaesthetized with 10 mM tetramisole and mounted on 2% agarose pads. Embryos were similarly mounted on agarose pads. Imaging was performed at room temperature (20–22°C) using an Olympus 60× 1.42 NA PLAPON Apochromat objective and 1.6× optovar on the set-up described above. For examination of neuronal morphology and recruitment/loss of centriolar and ciliary markers, 3D wide-field datasets were acquired, deconvolved, and processed as described above. Exposure conditions and scaling were kept constant when comparing signal across different developmental stages. For time-lapse imaging of neurite extension, 0.6-µm z stacks of GFP and single-plane differential interference contrast (DIC) images of embryos expressing myristoylated GFP under a panneuronal promoter were acquired every 5 min, using low-power LED illumination to avoid photodamage. GFP z stacks were maximum intensity projected in SoftWorx (Applied Precision), and image sequences were imported into ImageJ (National Institutes of Health) for video preparation.

### Electron microscopy and tomography

L4-stage worms were prepared by chemical fixation as previously described (Serwas and Dammermann, 2015). In brief, worms were fixed in 2.5% glutaraldehyde in cytoskeleton buffer (100 mM methyl ester sulfonate, 150 mM NaCl, 5 mM EGTA, 5 mM MgCl_2_, and 5 mM glucose in ddH_2_O, pH 6.1) overnight at 4°C. Samples were washed three times in the same buffer, postfixed for 30 min in 0.5% osmium tetroxide in buffer, and washed three times in buffer and once in ddH_2_O. Finally, samples were dehydrated for 15 min each in 40, 60, and 80%, twice in 95%, and three times in 100% acetone. L1-stage worms were fixed by high-pressure freezing (HPF Compact 01; M. Wohlwend GmbH) and freeze substitution (EM AFS2; Leica Biosystems) using 5% BSA in M9 buffer as filler and an acetone-based freeze substitution medium containing 0.5% glutaraldehyde, 0.5% osmium tetroxide, and 0.2% uranyl acetate. Freeze substitution was started at −140°C, and then temperature was gradually increased to −90°C over 3 h, kept at −90°C for 61 h, gradually increased to −30°C over 20 h, kept at −30°C for 12 h, and finally increased to 0°C over 15 h. Embryos were prepared according to the procedure described by Kolotuev et al. (2010). In brief, adult worms expressing myristoylated GFP under a panneuronal promoter were dissected in 2 µl M9 buffer on an Aclar film (no. 50425; Electron Microscopy Sciences) placed on a glass microscope slide. 30 µl of 4% low–melting point agarose (no. 0815; Amresco) was added, and the sample sandwiched between a second Aclar film and glass slide using an additional Aclar film piece containing a rectangular hole (∼3 × 1.5 cm) as a spacer. Embryos were excised using a biopsy punch and imaged by light microscopy to determine their developmental stage based on overall morphology by DIC and neuronal morphology by GFP fluorescence. Embryos of the desired stage were selected and fixed using high-pressure freezing (EM PACT; Leica Biosystems) and freeze substitution in 0.5% glutaraldehyde, 1% osmium tetroxide, and 0.2% uranyl acetate in acetone using the same time scheme as above. All samples were embedded in Agar100 resin after fixation and dehydration. For the data presented in Fig. 1 B, 70-nm serial sections were poststained with aqueous uranyl acetate and lead citrate and examined with a Morgagni 268D microscope (FEI) equipped with an 11-megapixel Morada CCD camera (Olympus) and operated at 80 kV. For all other experiments, 200-nm serial sections were poststained as above and examined with a Tecnai G2 20 microscope (FEI) equipped with an Eagle 4k HS CCD camera (FEI) and operated at 200 kV. Tilt series were acquired in single- or double-tilt axis geometry by stepwise tilting the sample from −60° to 60° in 1° increments using SerialEM (Mastronarde, 2005). Tomogram reconstruction with 10 nm gold beads as fiducials and model generation was performed using IMOD (Kremer et al., 1996). Tomographic slices presented a range from 10 to 25 nm in thickness.

### Statistical analysis

No statistical method was used to predetermine sample size. For fluorescence microscopy data, reproducibility was confirmed in at least two independent experiments. For electron microscopy data, the number of animals examined is provided in Table S2. Statistical significant differences between samples were determined by applying Student’s *t* test in Prism (GraphPad Software). Data distribution was assumed to be normal, but this was not formally tested. Two-tailed p-values <0.05 were considered significant. All results are expressed as the mean ± 95% confidence intervals. Sample size (*n*) and p-values are indicated in the figure legends.

### Online supplemental material

Fig. S1 examines functionality of fluorescent reporters used in this study. Fig. S2 examines loss of dynamically localized centriolar components during *C. elegans* ciliogenesis. Fig. S3 examines the role of DYF-19 in cilium assembly and maintenance. Table S1 lists strains used in this study. Table S2 lists numbers of animals examined by electron microscopy. Video 1 shows an electron tomogram and 3D model of the ciliary base in L1-stage larva (related to Fig. 1 E). Video 2 examines the ciliary base in chemically fixed L4-stage larva (related to Fig. 1 E). Video 3 shows neuronal development in late-stage *C. elegans* embryo (related to Fig. 2 A). Video 4 examines centriole ultrastructure in comma-stage embryo (related to Fig. 2 B). Video 5 examines docked centriole in twofold-stage embryo (related to Fig. 3 D). Video 6 examines the amphid channel and cilium in L1-stage larva (related to Fig. 4 C).

## Supplementary Material

Supplemental Materials (PDF)

Video 1

Video 2

Video 3

Video 4

Video 5

Video 6
