# Supplementary material for: Centrioles initiate cilia assembly but are dispensable for maturation and maintenance in C. elegans
Source: J Cell Biol. 2017 Jun 5;216(6):1659–71. doi: 10.1083/jcb.201610070 (PMC5461022; doi:10.1083/jcb.201610070)
Supplement: Supplemental Materials (PDF) [file JCB_201610070_sm.pdf]

Serwas et al., https://doi.org/10.1083/jcb.201610070

THE JOURNAL OF CELL BIOLOGY

A

Embryonic viability

Wild-type	98.4% (n=1523)
sas-6(ok2554) + SAS-6:GFP	98.7% (n=1244)
sas-4(tm3951) + GFP:SAS-4	95.3% (n=997)

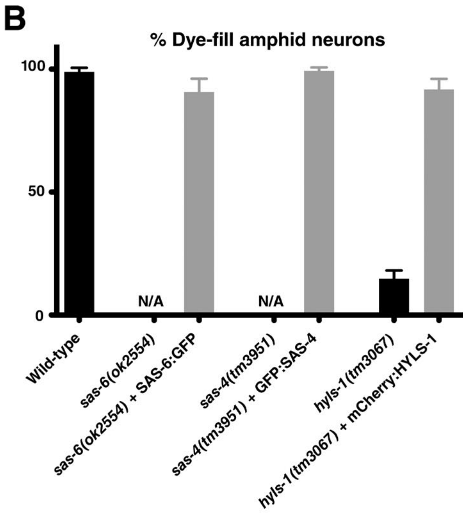

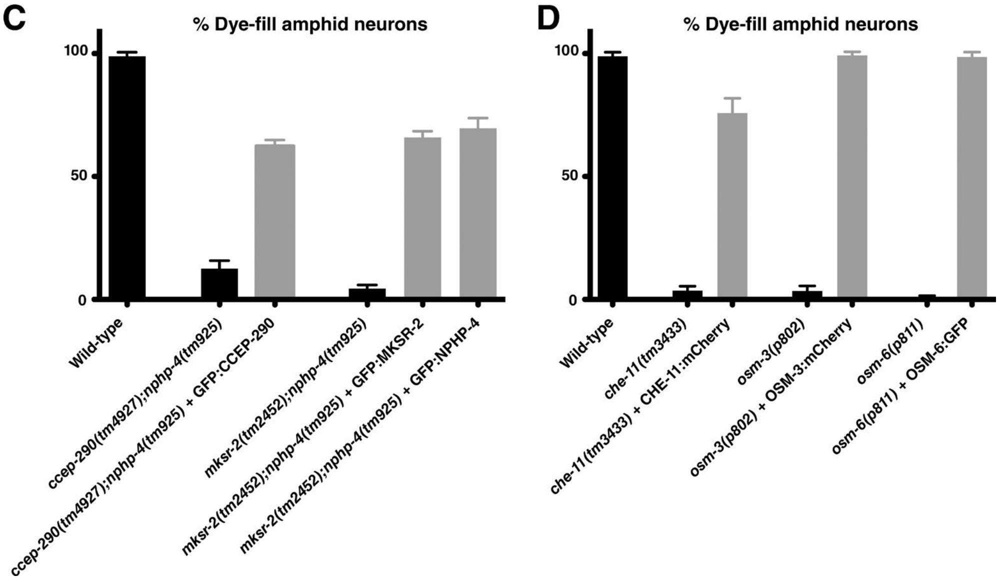

Figure S1. **Functionality of fluorescent fusions.** (A) Embryonic viability of *sas-6*/*sas-4* deletion mutants rescued by endogenous promoter GFP fusions compared with N2 wild type. *sas-6* and *sas-4* mutants exhibit fully penetrant maternal effect sterility in the absence of a functional copy of the gene (Song et al., 2011). Both transgenes fully restore viability. (B) Amphid cilia structural integrity in centriolar mutants is rescued by endogenous promoter GFP/mCherry fusions as assessed by dye filling. 12 amphid neurons consistently dye fill with DiI in wild type, comprising the bilaterally symmetric pairs of ASH, ASI, ASJ, ASK, ADL, and AWB neurons (Starich et al., 1995). Dye-fill phenotype expressed as percentage of wild-type complement is shown. Error bars are the 95% confidence interval. $n > 50$ animals assessed per condition. All three fluorescent fusions sustain cilia assembly in the absence of the endogenous protein. *hyls-1*–mutant data from Fig. 5 C are shown for comparison. (C) Amphid cilia structural integrity in transition zone mutants rescued by endogenous promoter GFP fusions, assessed by dye-filling as in B. Note that transition zone mutants display synthetic dye-fill phenotypes in double mutants combining components of the NPHP and either MKS or CEP290 modules (Schouteden et al., 2015). Defects are rescued by expression of either component tagged with GFP. Wild-type data are shown for comparison. (D) Amphid cilia structural integrity in IFT mutants rescued by endogenous promoter GFP/mCherry fusions, assessed by dye filling as in B. All three fluorescent fusions sustain cilia assembly in the absence of the endogenous protein. (B–D) All mutant rescues are statistically significant (Student's *t* test; $P < 0.0001$).

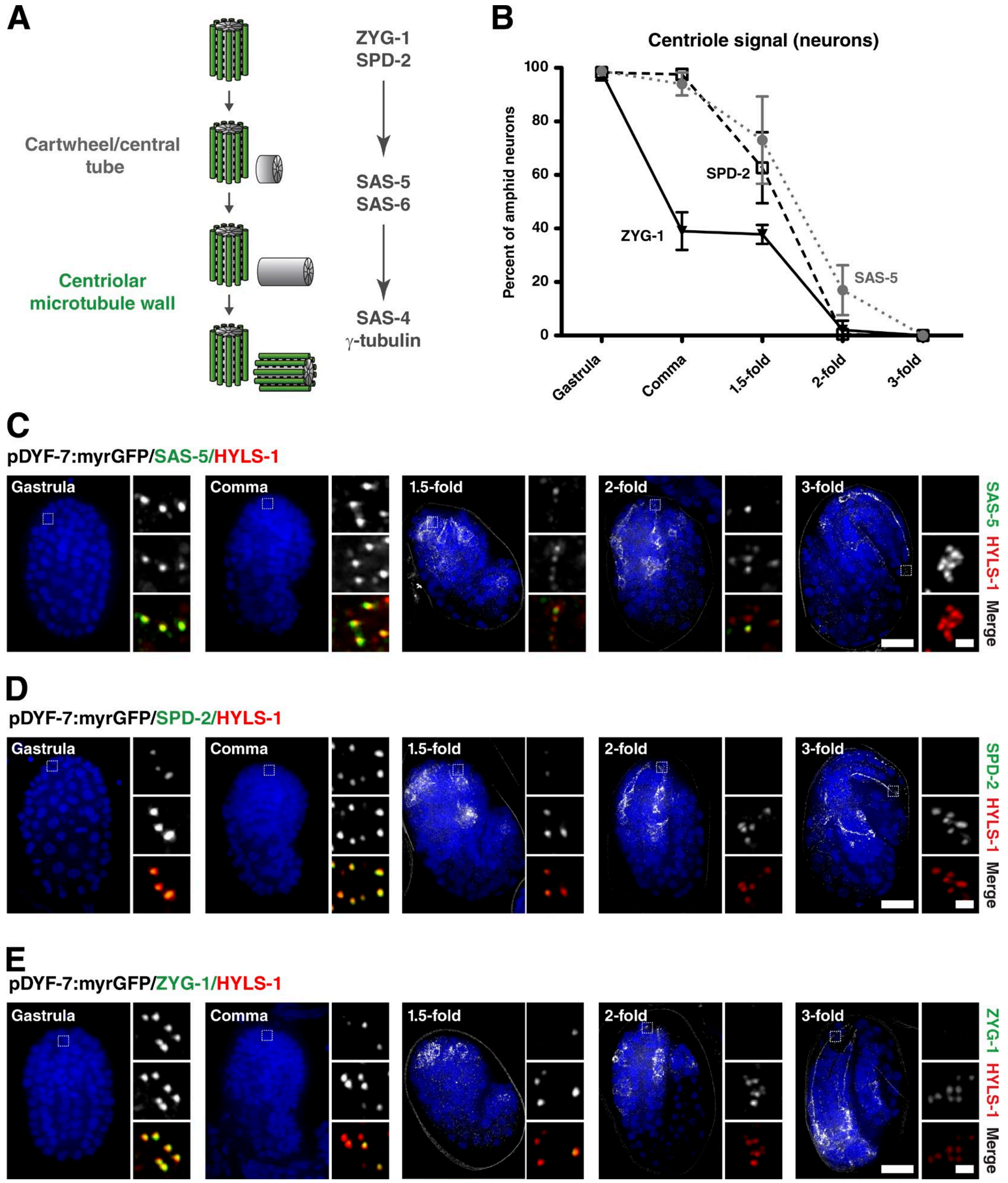

Figure S2. **Further characterization of centriole loss.** (A) Centriole assembly pathway as delineated in *C. elegans* (Delattre et al., 2006; Pelletier et al., 2006; Dammermann et al., 2008). Proteins act in a hierarchical manner, with SPD-2 and ZYG-1 promoting the recruitment of SAS-5 and SAS-6 to form the ninefold symmetric scaffold structure of the cartwheel or central tube. SAS-4 together with γ-tubulin then directs assembly of the centriolar microtubule wall. (B) Quantitation of centriolar signal in amphid neurons (percentage of HYLS-1 foci positive for ZYG-1, SPD-2, or SAS-5) from images as in C–E. Error bars are 95% confidence intervals. $n = 7$ animals per condition. (C–E) Immunofluorescence micrographs of gastrula-, comma-, 1.5-fold–, twofold-, and threefold-stage embryos expressing myristoylated GFP in amphid neurons and stained for HYLS-1 and SAS-5 (C), SPD-2 (D), or ZYG-1 (E). Insets show magnified view of ciliary base. Centriolar signal is lost from the ciliary base, with loss of ZYG-1 preceding that of SPD-2 and SAS-5. All three proteins are lost in nonneuronal cells coinciding with terminal differentiation. Bars: (C–E) 10 µm; (insets) 1 µm.

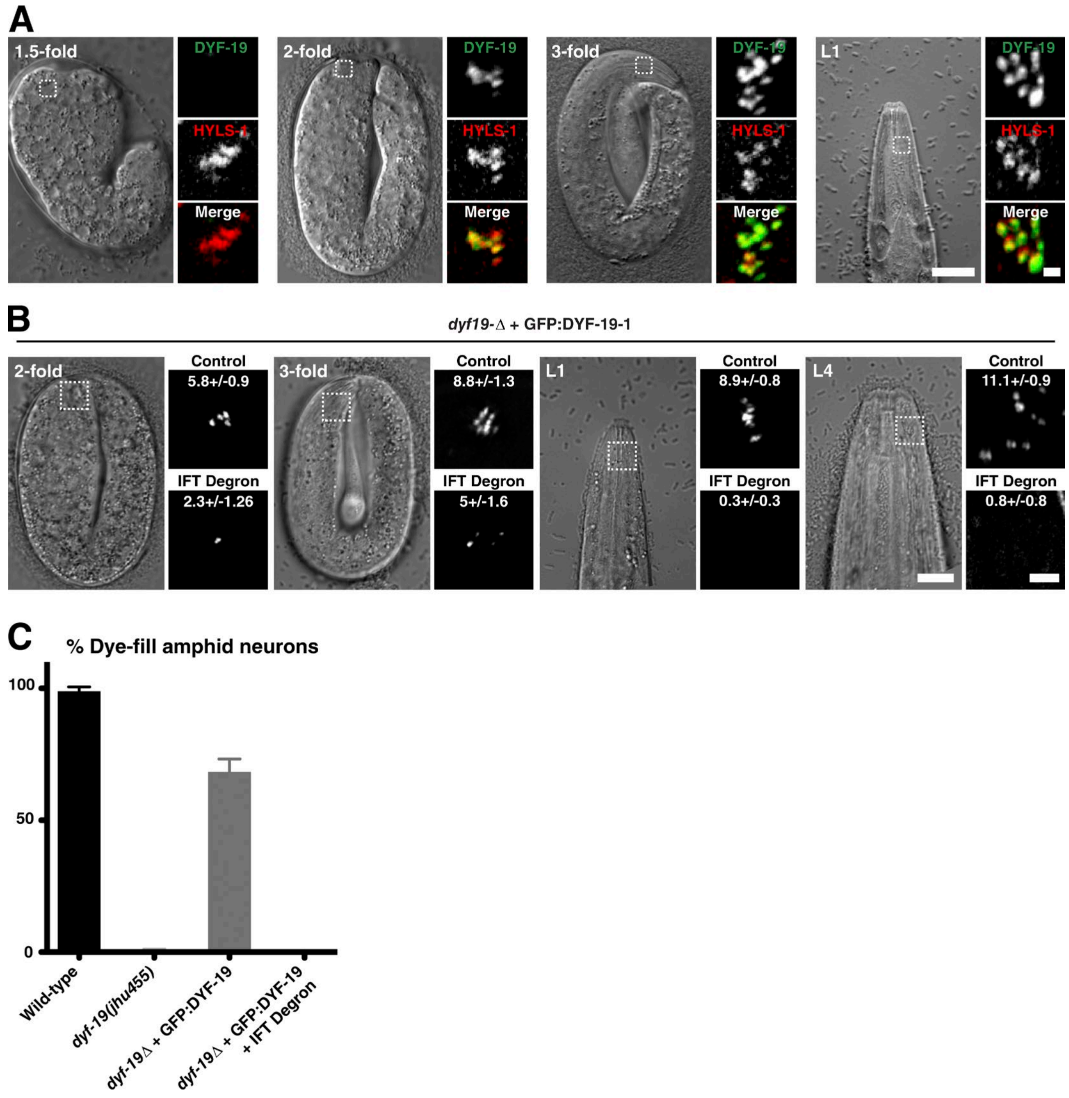

Figure S3. **Cilia maintenance requires DYF-19.** (A) Recruitment of the IFT docking factor DYF-19 to the site of cilia assembly marked by HYLS-1. Panels show 1.5-fold–, twofold-, and threefold-stage embryos and L1-stage larvae coexpressing GFP:DYF-19 and mCherry:HYLS-1. Recruitment occurs at the twofold stage, coincident with the onset of IFT accumulation (compare with Fig. 4). (B) Degron-mediated degradation results in a transient accumulation of GFP:DYF-19. All signal is lost by the L1 stage. Control animals not expressing degron are shown for comparison. Number of foci/amphid bundle ± 95% confidence intervals (n = 10–12 amphids per condition) is shown. Bars: (A and B) 10 µm; (insets) 2 µm. (C) Amphid cilia structural integrity as assessed by dye filling. Dye-fill phenotype expressed as percentage of wild-type complement is shown. *dyf-19* mutants display strong defects because of compromised axoneme assembly. These are rescued by expression of GFP:DYF-19 (Student's t test; $P < 0.0001$). Degron-mediated degradation of GFP:DYF-19 after onset of ciliogenesis abolishes dye filling (Student's t test; $P < 0.0001$). Wild-type data are shown for comparison. Error bars are 95% confidence interval. $n > 80$ animals per condition.

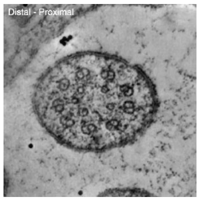

Video 1. **Electron tomogram and 3D model of the ciliary base in L1-stage larva.** Z scan through 130 nm of the ciliary base beginning in the transition zone and 3D reconstruction model highlighting ciliary membrane (gray) and doublet microtubules (green). Dual-axis tilt series was acquired on a Tecnai G2 20 microscope (FEI). Tomogram reconstruction was performed using IMOD.

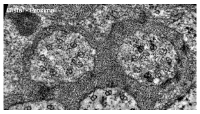

Video 2. **Electron tomogram and 3D model of ciliary base in chemically fixed L4-stage larva.** Z scan through 90 nm of the ciliary base beginning in the transition zone and 3D reconstruction model highlighting ciliary membrane (gray) and doublet microtubules (green). Dual-axis tilt series was acquired on a Tecnai G2 20 microscope (FEI). Tomogram reconstruction was performed using IMOD.

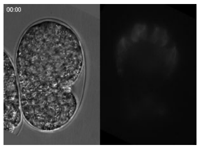

Video 3. **Neuronal development in late-stage *C. elegans* embryo.** Time-lapse sequence of an embryo expressing myristoylated GFP in amphid neurons beginning at the comma stage and continuing until the threefold stage. Single-plane GFP and transmitted light images were acquired every 5 min on a wide-field deconvolution microscope (DeltaVision; Applied Precision). The video playback is eight frames per second. Retrograde extension of dendrites begins at ~30 min, with cell bodies moving posterior, whereas dendritic tips remain fixed in position. At ~1 h 40 min, the worm begins to move inside the egg because of muscle contraction.

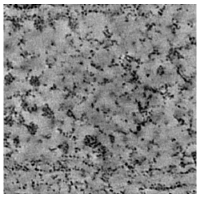

Video 4. **Electron tomogram and 3D model of the centriole in a comma-stage embryo.** Z scan through 85 nm of a centriole and 3D reconstruction model highlighting doublet microtubules (green). Dual-axis tilt series acquired on a Tecnai G2 20 microscope (FEI). Tomogram reconstruction was performed using IMOD.

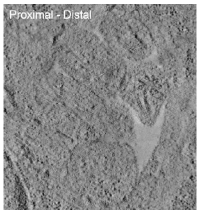

Video 5. **Electron tomogram and 3D model of docked centriole in a twofold-stage embryo.** Z scan through 150 nm of the amphid channel, zooming in on a docked centriole with a 3D reconstruction model highlighting ciliary membrane (gray), doublet microtubules (green), transition zone Y-links (red), and the central cylinder (brown). Parts of a second centriole, also composed of doublet microtubules and oriented orthogonally, are also captured in the section. Single-axis tilt series was acquired on a Tecnai G2 20 microscope (FEI). Tomogram reconstruction was performed using IMOD.

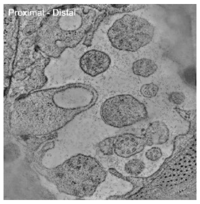

Video 6. **Electron tomogram and 3D model of cilium in L1-stage larva.** Z scan through 110 nm of the base of the amphid channel, zooming in on cilium with a 3D reconstruction model highlighting the ciliary membrane (gray) and doublet microtubules (green). Dual-axis tilt series was acquired on a Tecnai G2 20 microscope (FEI). Tomogram reconstruction was performed using IMOD.

Table S1. ***C. elegans* strains used in this study**

Strain	Genotype
N2	*wild-type*
DAM131	*mksr-2(tm2452)IV; nphp-4(tm925)V*
DAM181	*vieSi18[pAD154; Psas-4::gfp::sas-4reencoded; cb unc-119(+)]; sas-4(tm3951)II*
DAM276	*ltSi40[pOD1227; Psas-6::sas-6reencoded::gfp; cb unc-119(+)]II; sas-6(ok2554)IV*
DAM305	*ccep-290(tm4927)I; nphp-4(tm925)V*
DAM306	*ccep-290(tm4927)I; vieSi12[pAD373; Pccep-290::gfp::ccep-290cDNA; cb unc-119(+)]II; nphp-4(tm925)V*
DAM318	*vieSi16[pAD390; Phyls1::mcherry::hyls-1; cb unc-119(+)]IV; hyls-1(tm3067)V*
DAM455	*vieSi22[pAD401; Pmksr-2::gfp::mksr-2; cb unc-119(+)]I; vieSi16[pAD390; Phyls1::mcherry::hyls-1; cb unc-119(+)]IV*
DAM456	*vieSi12[pAD373; Pccep-290::gfp::ccep-290cDNA; cb unc-119(+)]II, vieSi16[pAD390; Phyls-1::mcherry::hyls-1; cb unc-119(+)]IV*
DAM459	*vieSi23[pAD402; Pnphp-4:gfp::nphp-4cDNA; cb unc-119(+)]I; vieSi16[pAD390; Phyls-1::mcherry::hyls-1; cb unc-119(+)]IV*
DAM488	*vieSi22[pAD401; Pmksr-2::gfp::mksr-2; cb unc-119(+)]I; mksr-2(tm2452)IV; nphp-4(tm925)V*
DAM489	*vieSi23[pAD402; Pnphp-4::gfp::nphp-4cDNA; cb unc-119(+)]I; mksr-2(tm2452)IV; nphp-4(tm925)V*
DAM801	*vieEx30[Pdyf-7::myristyl-gfp; rol-6(su10060)]*
DAM835	*che-11(tm3433)V*
DAM837	*vieSi22[pAD401; Pmksr-2::gfp::mksr-2; cb unc-119(+)]I; vuaSi2[pBP22; Posm-3::osm-3::mcherry; cb unc-119(+)]II; osm-3(p802)IV*
DAM838	*vieSi22[pAD401; Pmksr-2::gfp::mksr-2; cb unc-119(+)]I; vuaSi24[pBP43; Pche-11::che-11::mcherry; cb unc-119(+)]II; che-11(tm3433)V*
DAM839	*vuaSi15[pBP36; Posm-6::osm-6::gfp; cb unc-119(+)]I; vuaSi21[pBP39; Pmks-6::mks-6::mcherry; cb unc-119(+)]II; osm-6(p811)V*
DAM840	*ltSi914[pOD2048; Posm-6::vhhgfp4::zif-1::operon-linker::mcherry::his-11::tbb-2 3′UTR; cb-unc-119(+)]II; ltIs67[pOD348; Ppie-1::gfp::hyls-1 cDNA; unc-119 (+)]IV; hyls-1(tm3067)V*
DAM841	*vieSi18[pAD154; Psas-4::gfp::sas-4reencoded; cb unc-119(+)]II; sas-4(tm3951)III; vieSi16[pAD390; Phyls1::mcherry::hyls-1; cb unc-119(+)]IV*
DAM842	*ltSi40[pOD1227; Psas-6::sas-6reencoded::gfp; cb unc-119(+)]II; sas-6(ok2554); vieSi16[pAD390; Phyls1::mcherry::hyls-1; cb unc-119(+)]IV*
DAM880	*vieSi64[pAD672; Pccep-290::mNeonGreen::ccep-290; cb unc-119(+)]I; vieSi70[pAD675; Pche-11::che-11::mkate2]IV*
DAM882	*vieSi64[pAD672; Pccep-290::mNeonGreen::ccep-290; cb unc-119(+)]I; vieSi70[pAD675; Pche-11::che-11::mkate2]IV; hyls-1(tm3067)V*
DAM883	*vieSi64[pAD672; Pccep-290::mNeonGreen::ccep-290; cb unc-119(+)]I; ltSi914[pOD2048; Posm-6::vhhgfp4::zif-1::operon-linker::mcherry::his-11::tbb-2 3′UTR; cb-unc-119(+)]II; vieSi72[pAD673; Pdyf-19::mkate2::dyf-19]; ltIs67[pOD348; Ppie-1::gfp::hyls-1 cDNA; unc-119 (+)]IV; hyls-1(tm3067)V*
DAM887	*vieSi64[pAD672; Pccep-290::mNeonGreen::ccep-290; cb unc-119(+)]I;ltSi914[pOD2048; Posm-6::vhhgfp4::zif-1::operon-linker::mcherry::his-11::tbb-2 3′UTR; cb-unc-119(+)]II; vieSi70[pAD675; Pche-11::che-11::mkate2]; ltIs67[pOD348; Ppie-1::gfp::hyls-1 cDNA; unc-119 (+)]IV; hyls-1(tm3067)V*
DAM889	*vieSi64[pAD672; Pccep-290::mNeonGreen::ccep-290; cb unc-119(+)]I; vieSi72[pAD673; Pdyf-19::mkate2::dyf-19]IV*
DAM890	*vieSi64[pAD672; Pccep-290::mNeonGreen::ccep-290; cb unc-119(+)]I; vieSi72[pAD673; Pdyf-19::mkate2::dyf-19]IV; hyls-1(tm3067)V*
DAM893	*vieSi16[pAD390; Phyls-1::mcherry::hyls-1; cb unc-119(+)];vieSi73[pAD687; pdyf-19::gfp::dyf-19; cb unc-119(+)]IV*
DAM947	*dyf-19(jhu455)V*
DAM949	*vieSi73[pAD687; pdyf-19::gfp::dyf-19; cb unc-119(+)]IV; dyf-19(jhu455)V*
DAM963	*ltSi914[pOD2048; Posm-6::vhhgfp4::zif-1::operon-linker::mcherry::his-11::tbb-2 3′UTR; cb-unc-119(+)]II; vieSi73[pAD687; pdyf-19::gfp::dyf-19; cb unc-119(+)]IV; dyf-19(jhu455)V*
EJP16	*vuaSi2[pBP22; Posm-3::osm-3::mcherry; cb unc-119(+)]II; unc-119(ed3) III; osm-3(p802)IV*
EJP70	*mks-6(gk674)I; vuaSi21[pBP39; Pmks-6::mks-6::mcherry; cb unc-119(+)]II; kap-1(ok676)III; vuaSi1[pBP20; Pkap-1::kap-1::gfp; cb unc-119(+)]IV*
EJP76	*vuaSi15[pBP36; Posm-6::osm-6::gfp; cb unc-119(+)]I; osm-6(p811)V*
EJP81	*vuaSi24[pBP43; Pche-11::che-11::mcherry; cb unc-119(+)]II; che-11(tm3433)V*
OD192	*hyls-1(tm3067)V*
OD199	*ltIs67[pOD348; Ppie-1::gfp::hyls-1 cDNA; unc-119 (+)]IV; hyls-1(tm3067)V*

Table S2. **Number of animals examined by transmission electron microscopy/electron tomography**

Strain	Description	Stage	Type of fixation	Figure	Number of animals
N2	Wild-type	L4	Chemical	Figs. 1 B and 5 F; Video 2	>5
N2	Wild-type	L1	HPF/FS	Figs. 1 E and 4 C; Videos 1 and 6	3
DAM801	pDYF-7:myrGFP	Comma	HPF/FS	Fig. 2 B; Video 4	3
DAM801	pDYF-7:myrGFP	2-fold	HPF/FS	Fig. 3 D; Video 5	3
OD192	*hyls-1* mutant	L4	Chemical	Fig. 5 F	5
DAM840	*hyls-1* mutant + GFP:HYLS-1 + IFT degron	L4	Chemical	Fig. 5 F	3

References

Dammermann, A., P.S. Maddox, A. Desai, and K. Oegema. 2008. SAS-4 is recruited to a dynamic structure in newly forming centrioles that is stabilized by the γ-tubulin–mediated addition of centriolar microtubules. *J. Cell Biol.* 180:771–785. http://dx.doi.org/10.1083/jcb.200709102

Delattre, M., C. Canard, and P. Gönczy. 2006. Sequential protein recruitment in C. elegans centriole formation. *Curr. Biol.* 16:1844–1849. http://dx.doi.org/10.1016/j.cub.2006.07.059

Pelletier, L., E. O'Toole, A. Schwager, A.A. Hyman, and T. Müller-Reichert. 2006. Centriole assembly in *Caenorhabditis elegans*. *Nature.* 444:619–623. http://dx.doi.org/10.1038/nature05318

Schouteden, C., D. Serwas, M. Palfy, and A. Dammermann. 2015. The ciliary transition zone functions in cell adhesion but is dispensable for axoneme assembly in *C. elegans*. *J. Cell Biol.* 210:35–44. http://dx.doi.org/10.1083/jcb.201501013

Song, M.H., Y. Liu, D.E. Anderson, W.J. Jahng, and K.F. O'Connell. 2011. Protein phosphatase 2A-SUR-6/B55 regulates centriole duplication in C. elegans by controlling the levels of centriole assembly factors. *Dev. Cell.* 20:563–571. http://dx.doi.org/10.1016/j.devcel.2011.03.007

Starich, T.A., R.K. Herman, C.K. Kari, W.H. Yeh, W.S. Schackwitz, M.W. Schuyler, J. Collet, J.H. Thomas, and D.L. Riddle. 1995. Mutations affecting the chemosensory neurons of Caenorhabditis elegans. *Genetics.* 139:171–188.
